# Boar Semen Shipping for Artificial Insemination: Current Status and Analysis of Transport Conditions with a Major Focus on Vibration Emissions

**DOI:** 10.3390/ani12101331

**Published:** 2022-05-23

**Authors:** Tim Hafemeister, Paul Schulze, Ralf Bortfeldt, Christian Simmet, Markus Jung, Frank Fuchs-Kittowski, Martin Schulze

**Affiliations:** 1 Institute for Reproduction of Farm Animals Schönow, D-16321 Bernau, Germany; t.hafemeister@ifn-schoenow.de (T.H.); r.bortfeldt@ifn-schoenow.de (R.B.); m.jung@ifn-schoenow.de (M.J.); 2 IFN Schönow GmbH, D-16321 Bernau, Germany; 3 Environmental Informatics, Campus Wilhelminenhof, University of Applied Science, D-10313 Berlin, Germany; paul.schulze@htw-berlin.de (P.S.); frank.fuchs-kittowski@htw-berlin.de (F.F.-K.); 4 Minitüb GmbH, D-84184 Tiefenbach, Germany; csimmet@minitube.de

**Keywords:** artificial insemination, boar semen, transport, vibrations, quality assurance

## Abstract

**Simple Summary:**

Liquid preserved boar semen is a perishable product, and many environmental influences can affect sperm quality during transport from the boar station to the sow farm. Previous studies have shown that vibration emissions have a negative effect on spermatozoa, however, there has been no documentation of the vibrations spermatozoa are exposed to during transport. To answer this question, several breeding companies worldwide were interviewed about their delivery process. As it turns out, environmental influences are only rarely recorded, and vibrations are not monitored at all. To record and to evaluate vibrations that occur during transport, we have developed a measurement system and investigated vibration intensities in standardized road trials. We were able to show that both speed and road surface significantly influence the produced vibrations. Furthermore, the occurrence of the vibrations on a reference delivery from a German boar stud was studied. In the future, it is recommended that in order to avoid negative influences during transport, the transport of boar semen should be permanently monitored.

**Abstract:**

In the modern pig reproduction system, artificial insemination (AI) doses are delivered from AI centers to sow farms via logistics vehicles. In this study, six breeding companies in three countries (Brazil, Germany, and the USA) were interviewed about their delivery process. It was found that there is currently no comprehensive monitoring system for the delivery of semen. The entire process “shipping of boar semen” was documented using Business Process Model and Notation (BPMN). Although it is not currently known which vibrations occur at all, it is suspected that vibration emissions affect the quality of boar semen. For this reason, a prototype of a measuring system was developed to calculate a displacement index (*D_i_*), representing vibration intensities. Vibrations were analyzed in standardized road trials (*n* = 120) on several road types (A: smooth asphalt pavement, B: rough asphalt pavement, C: cobblestone, and D: dirt road) with different speeds (30, 60, 90, 120, and 150 km/h). A two-way ANOVA showed significant differences in mean *D_i_*, depending on road surface and speed as well as an interaction of both factors (*p* < 0.001). A field study on a reference delivery from a German AI center to several sow farms indicated that 33% of the observed roads are in good quality and generate only a few vibrations (*D_i_* ≤ 1), while 40% are of a moderate quality with interrupted surfaces (*D_i_* = 1–1.5). However, 25% of the roads show markedly increased vibrations (*D_i_* ≥ 1.5), as a consequence of bad conditions on cobblestones or unpaved roads. Overall, more attention should be paid to factors affecting sperm quality during transport. In the future, an Internet of Things (IoT) based solution could enable complete monitoring of the entire transport process in real time, which could influence the courier’s driving behavior based on road conditions in order to maintain the quality of the transported AI doses.

## 1. Introduction

Over the last decades, artificial insemination (AI) has become the most important biotechnology in modern pig reproduction systems [1] and many efforts have been made to provide liquid extended boar semen of the highest quality. Quality assurance (QA) programs aiming at boar selection, sperm quality, hygienic critical control points, temperature regime, and even correct extender preparation have been developed [2]. As a part of the production chain, AI doses are delivered from AI centers to sow farms via logistics vehicles, either to be used for immediate insemination or within a few days. Although this step in the production chain could significantly influence sperm quality, little is known about what happens during transport. Potential influencing factors previously identified are temperature deviations and UV light [3], however, new research results show that vibration emissions may also affect sperm quality [4]. This effect depends on several factors such as: frequency and duration of vibrations, type of semen extender, and air volume in the AI dose as well as temperature during transport [5].

Numerous studies have investigated the effects of vibrations on other transported goods such as fruits [6,7] or drinks [8]. For instance, transport-related losses in apples are estimated at 15% [9]. Furthermore, paintings [10] or electronic goods like lithium-ion cells [11] have to be protected against vibrations and shocks during transport. The health effects of whole-body vibrations on professional drivers, such as lower back pain, have been studied for a long time [12], and limits of daily exposure were set in many countries to increase occupational health and safety [13]. Even animal welfare has been found to be affected by vibrations during transport [14]. However, further research found that international test standards for vibration and shock events do not reflect vibrations actually occurring in real-time during transport [15]. Although there are new technical possibilities such as mobile sensing [2,16] or the Internet of Things [17], no comprehensive system currently exists to detect vibration emissions on transported AI doses. This study aims to describe the process of developing a new QA system for boar semen shipping, and it is divided into the following sections: current state of boar semen shipping (1), outlining the business process of boar semen shipping (2), and collection and analysis of vibration data under standardized (3) and field conditions (4).

## 2. Materials and Methods

### 2.1. Current State of Boar Semen Shipping

In the first step, the current state of boar semen shipping was evaluated to estimate requirements for a new QA system. To get a global comparison of different transport conditions, data on common delivery practices of six breeding companies in Brazil (*n* = 1), Germany (*n* = 4), and the USA (*n* = 1), housing approximately 10,000 AI boars in total were gathered by individual online interviews. This provided information regarding the shipping process, with a focus on packaging and monitoring. Information was mainly gathered on the act of transportation itself, however, data on general production and information technology-(IT)-support were also collected.

### 2.2. Business Process Documentation and Analysis of Boar Semen Shipping

In order to obtain an overview of the conditions boar semen are exposed to during transport, data was documented and analyzed using process descriptions and process models. Identified processes were modeled using Business Process Model and Notation (BPMN); BPMN is a modelling language used to graphically model and document business processes [18]. For this purpose, the entire process is divided into subtasks, which are each described using activities.

### 2.3. Collection and Analysis of Vibration Data during the Transport of Boar Semen Doses under Standardized and Field Conditions

To measure the environmental impacts of transport-related influences, we developed a measurement system consisting of a data logger and a smartphone application. The goal was to develop a flexible and an easy-to-program system, which was compatible with a variety of different sensors. The data logger consists of a Nano 33 BLE microcontroller from Arduino, and it is equipped with multiple sensors that measure three-axis acceleration, temperature, air pressure, light intensity, and relative humidity. It also comes equipped with a wireless connector. Arduino is an open source platform consisting of hardware and software components [19]. The hardware component consists of development boards with a microcontroller as the computing unit and several analog and digital inputs and outputs. On the software side, the platform consists of a development environment that adds an abstraction layer for programming the microcontroller. The wireless connection between the data logger and the smartphone is established via Bluetooth Low Energy (BLE). This connection is robust and fast, and it allows a link-up, which is easier to establish than when using Bluetooth Classic or WiFi [20]. An Android application was developed for controlling the microcontroller as well as displaying and storing the measured data. With this application, the measured data of the data logger is stored locally on the smartphone in a text file using key-value pairs. The duration of the recording can be set in advance. The speed and the current road type as well as individual events, such as hard braking or potholes, can be logged to create a temporal link within the measured data. In addition, the date, time, and geolocation data (GNSS) of the smartphone are stored. During measurements in the vehicle, the data logger was supplied with power via a vehicular outlet. Processing of the raw data was performed by a Perl script that calculated the distance *D* as vector length between two measurement points *p_m_* and *p_n_* (Equation (1)):

(1) $$D=\left|\vec{p_{m}p_{n}}\right|=\sqrt{{\left(x_{n}-x_{m}\right)}^{2}+{\left(y_{n}-y_{m}\right)}^{2}+{\left(z_{n}-z_{m}\right)}^{2}}$$

These measurement points are described by their tri-axial acceleration data (*x*, *y*, *z*) at contiguous points in time *m* and *n*, representing consecutive spatial points. Next, the displacement index *D_i_* is generated as an average data point per second to rate the intensity of the vibration (Equation (2)). It is combined from all calculated *D*-values of one second, depending on the sampling frequency *f*:

(2) $$D_{i}=\frac{\sum_{k=1}^{f}D_{k}}{f}$$

In order to estimate the occurring vibrations on different road surfaces, test drives took place on roads paved with smooth (A) and rough (B) asphalt, cobblestone (C), and dirt road (D). The rough asphalt pavement is said to have frequent interruptions, minor potholes, and bumps, while there are no such irregularities on smooth pavement. For category A and B, both country roads and motorways were driven. Different speeds were kept constant with the use of a cruise control system, and displacement data was recorded for *t* = 60 s with a sampling rate of *f* = 50 Hz. Different test groups containing routes with similar road surfaces and conditions were chosen, and several test drives were recorded. Ten sample measurements, without any discontinuity, were selected from each test group. The occurring vibration intensities were plotted in a time–displacement index diagram. To capture the sum of all single events of one test drive, the mean displacement index $D_{\bar{x}}$ for *t* = 60 s was considered and compared between the different test runs (Equation (3)):

(3) $$D_{\bar{x}}=\frac{\sum_{j=1}^{t}D_{i_{j}}}{t}$$

Due to possible loss of information during calculation of the mean displacement index $D_{\bar{x}}$, standard deviation and coefficient of variation were also calculated. Furthermore, minimum and maximum displacement indices were included in the analysis. For these standardized road trials, the measurement system was fastened in the trunk of a Volkswagen Caddy III, and every trial for road surface and speed was repeated ten times (*n* = 120 in total).

In order to determine the frequency of different vibration intensities affecting the delivered AI doses, an 800 km delivery of AI doses from a German boar stud was accompanied and the occurring vibrations were recorded during the entire trip. The measurement system was placed inside of an air-conditioned transport box (Minitüb, Tiefenbach, Germany), next to the transported AI doses. A Ford Transit Connect was used as a delivery vehicle for this trip.

### 2.4. Statistical Analysis

A two-way Analysis of Variance (ANOVA) was performed to evaluate the overall dependence of mean displacement index $D_{\bar{x}}$ on speed and on road surface, and a post hoc Tukey Honestly Significant Difference Test (Tukey’s HSD) identified significant differences between the individual test groups. A *p*-value of <0.05 was considered significant. Graphing and statistical analysis, including the prerequisite check for normal distribution and homogeneity of variance of the data, were carried out using the ggpubr package [21] and the statistical package of R [22].

## 3. Results

### 3.1. Current State of Boar Semen Shipping

The international comparison of general transport issues is summarized in Table 1. Boar semen is produced and packaged in a timely fashion, and it is intended for same day shipping. To avoid chilling injury [23], AI doses are slowly cooled down (≈4 °C per hour) in the boar stud or during transport. For biosecurity purposes, AI portions are generally packaged in plastic bags. Double-bagged packaging was only reported from one breeding company. Most respondents send their delivery directly from the AI center to the customers, while a minority first supply a central shipping station and then distribute the products from several studs at the same time. In addition, there are also sperm depots where customers can collect their products themselves. Commonly, either passenger or commercial vehicles operated by the company’s own couriers or subcontractors are used for transport. Other shipping service providers handle long-distance or international shipping. In the vehicles, AI doses are stored in air-conditioned boxes or, less commonly, in polystyrene boxes with cool or warm packs, depending on the outside temperature. One breeding company uses vehicles with air-conditioned trunks.

None of the interviewed breeding companies use a permanent monitoring system during transport. The temperature of the product can be manually measured or checked with integrated temperature sensors in the boxes. In part, the temperatures are documented by hand during the handover to the customer. Temperature loggers are sent at irregular intervals to record the temperature curve inside the boxes, and measured temperatures had a range from 1 °C to 28 °C. Except for relative humidity, which was mentioned by only one interviewee, no further parameters were routinely monitored. The couriers operating under contract to the AI centers received appropriate training regarding the handling of boar semen, especially in respect to the importance of temperature regulation. In the case of externally appointed shipping providers, proper handling cannot be guaranteed. Handling procedures designed by the AI centers require, for example, that transport of the AI doses from the vehicle to the recipient should occur in insulated containers. In addition, the vehicle engines should not be switched off during extreme temperatures in summer or in winter in order to keep the temperature in the vehicle constant. Furthermore, AI doses should be stored in the dark, however, none of the companies interviewed monitor this parameter. The influence of air pressure and relative humidity was addressed in the interviews; however, the AI centers considered these factors not to be relevant to the transport process as these parameters change only slowly and slightly.

Due to the lack of a monitoring system, none of the interviewed companies can evaluate the influence of temperature and vibration emissions on their vulnerable product. Notably, they showed great interest in IT-support regarding automatic documentation of the entire transport process as part of the complete production chain. There was agreement that temperature and vibration emissions should be monitored, as these can directly affect sperm quality.

### 3.2. Business Process Model and Notation: Shipping of Boar Semen Doses

**Figure 1:**
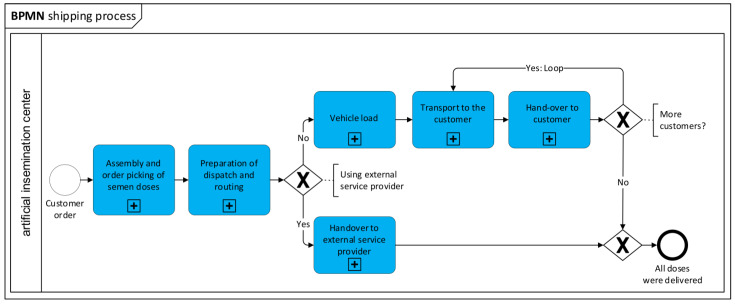
Overview of sub-processes of the business process “shipping of boar semen”, visualized using Business Process Model and Notation (BPMN).

The process of production and shipping boar semen from the AI center to the customer can be divided into several sub-processes: “customer order”, “assembly and order picking of semen doses”, “preparation of dispatch and routing”, “vehicle load”, “transport to customer”, “hand-over to customers”, “drive back to AI center”, and “hand-over at Al center”. If an external shipping provider is used, AI doses were handed over at the AI center or they were carried to the semen depot of a delivery service. The overall process of production and shipping is visualized as BPMN in Figure 1.

### 3.3. Analysis of Vibrations under Standardized Conditions

**Figure 2:**
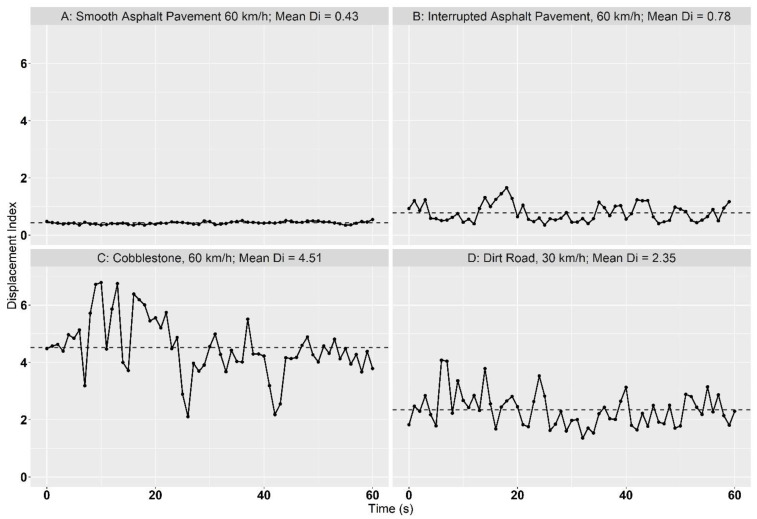
Exemplary Displacement Index curves for selected test runs. Dots on the chart line represent single measurement points of *D_i_*-values, and the dashed line shows the mean Displacement Index ($D_{\bar{x}}$) of this test run.

**Figure 3:**
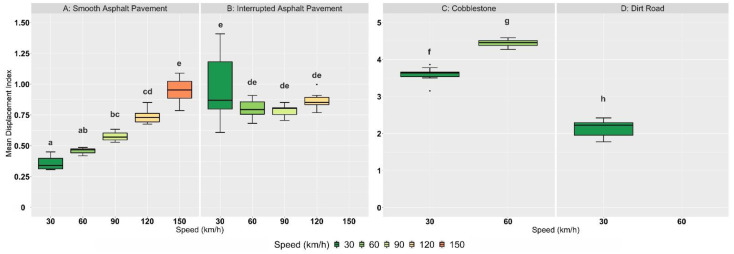
Summarized $D_{\bar{x}}$-values (mean Displacement Index *D_i_* for *t* = 60 s) from standardized road trials with one box per category (*n* = 120, 10 each category). Highest and lowest values are shown as whiskers, inter-quartiles between quartiles 1 and 3 depicted as boxes with a median line. Outliers draw dots. a–h: boxes with different letters differ significantly (*p* < 0.05).

Figure 2 shows the exemplary curves of the displacement index (*D_i_*) of some selected measured trials. Descriptive statistics of the standard road trial results are shown in Table 2 and contrasted in Figure 3.

The highest displacement values (both *D_i_* and $D_{\bar{x}}$) were recorded on cobblestone, with an increase in speed (30 km/h to 60 km/h) also causing an increase in the mean displacement index $D_{\bar{x}}$ (3.73 to 4.44). Individual data points, corresponding to one-second time intervals, even reached *D_i_*-values above six. Although not as pronounced as on cobblestone, the trend of higher $D_{\bar{x}}$-values with increasing speed can also be seen on roads paved with smooth asphalt. A different result is observed on rough asphalt, where it was difficult to find comparable tracks for each run. Irregularities in the road surface do not occur uniformly by definition, and not every route was suitable for measuring every defined speed class due to curves, speed limits, or traffic conditions. The mean $D_{\bar{x}}$-value at 30 km/h was higher than at other speed classes in this category, with a coefficient of variance of 26.8%, which is the highest of all groups. This indicates a large difference between the individual trials at this speed. While collecting displacement data on a dirt road (an unpaved road with deep potholes), it was not possible to use the cruise control system. In that case, the speed was kept as constant as possible at 30 km/h to avoid car damage. The measured results rank between rough asphalt and cobblestone (mean $D_{\bar{x}}$ = 2.14).

A two-way ANOVA was performed to analyze the effect of speed and road surface on the mean displacement index $D_{\bar{x}}$. It revealed that there was a significant interaction between the effects of speed and road surface (F(4, 108) = 48.76, *p* < 0.001). Simple main effects analysis showed that speed as well as road surface did have a significant effect on mean displacement index (*p* < 0.001). The summarized ANOVA results are shown in Table 3. A post hoc Tukey HSD test compared the mean $D_{\bar{x}}$-values of the individual test groups, each differing in the factor speed or road surface, and the results are summarized in Table 4. Looking at different speed classes on the same road surface, there is a significant difference in vibration intensity when speed is increased on cobblestones (*p* < 0.001). A similar trend in the difference of vibration intensities can be observed on smooth asphalt, although differences in $D_{\bar{x}}$ are only significant at larger speed differences (*p* < 0.05). Vibration differences between 30 and 60 km/h (*p* = 0.877), 60 and 90 km/h (*p* = 0.678), and 90 and 120 km/h (*p* = 0.201) are not significant on this road surface. On rough asphalt, no significant differences were observed in the mean $D_{\bar{x}}$-values between all different speed classes (*p* > 0.05). Considering vibration intensities within the same speed level, significant differences can be observed between most road surface types (*p* < 0.05), with an exception of the highest speed level (A 120 vs. B 120: *p* = 0.579).

### 3.4. Analysis of Vibration Emissions under Field Conditions

**Figure 4:**
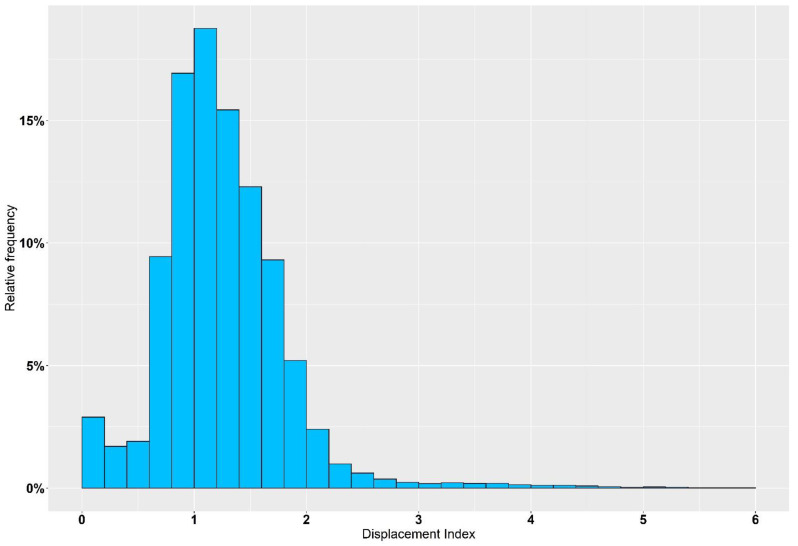
Summary of occurring Displacement Indices (*D_i_*) on a reference delivery from a German AI center to several sow farms (distance = 800 km, *t* = 590 min).

**Figure 5:**
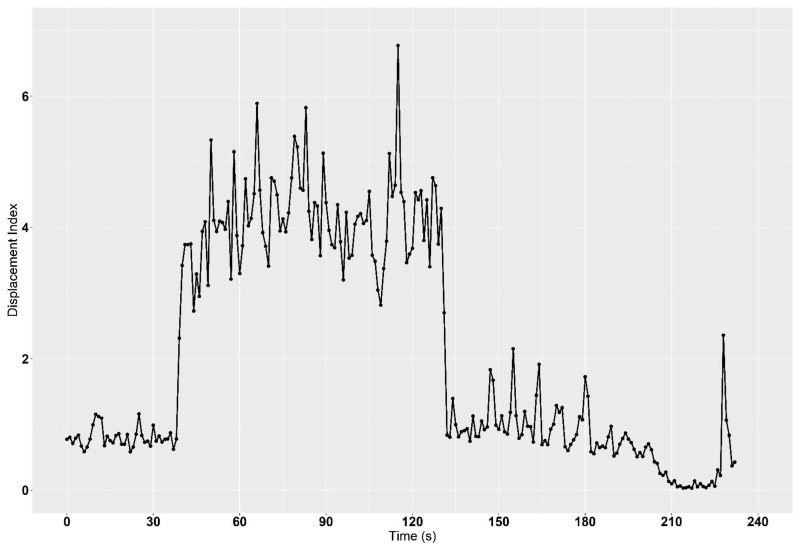
This small extract of a delivery (*t* = 232 s) shows an access road to a sow farm. On this road section, vibration emissions with a Displacement Index (*D_i_*) > 3 are recorded during a time span of 90 s. Dots on the chart line represent single measurement points of *D_i_*-values.

The mean displacement index ($D_{\bar{x}}$) over the entire trip of an 800 km reference delivery was 1.25 (*t* = 590 min), but to describe the occurring vibrations over the total duration, the relative frequency of different vibration intensities is more appropriate. Here it was found that *D_i_*-values between 1.0 and 1.2 were the most common with 18.8%, followed by 0.8–1.0 (16.9%) and 1.2–1.4 (15.4%). Summarized data are shown in Figure 4 and Table 5. Although over 90% of the determined *D_i_*-values under field conditions of boar semen transport are between 0 and 2, individual events with higher and partly heavy vibrations do appear. Especially, access roads to sow farms in rural areas, as shown in Figure 5, cannot be completely avoided.

## 4. Discussion

The main goal of the modern pig reproduction system is to increase the efficiency of artificial insemination [24]. One way of accomplishing this goal is to reduce the transport-related loss of boar sperm quality. Although there are many QA programs in the production chain of liquid extended boar semen [2], the deliveries are currently not monitored as standard. After analyzing the different transport conditions worldwide, it can be concluded that the most important factors affecting sperm quality are probably the different cooling systems and vehicle types as well as long transport durations. Data from experiments performed at the reference laboratory of the Institute for Reproduction of Farm Animals Schönow (IFN) in Germany, which measured handover temperatures, show that deviations from the recommended storage temperature for AI doses of between 16 and 18 °C [25] can occur regardless of transport container, especially when transport coincides with extreme seasonal temperatures. Transport time can vary greatly, and it was reported to take up to twelve hours; furthermore, if the product is first transported to a central depot to await further distribution, the transport times are likely even longer. This leads to an increased possibility of quality loss caused by transport-related influences. Due to very heterogeneous conditions, there will be no universal solution to handle different environmental extremes. Using a QA system to monitor all relevant parameters, as well as providing a warning in case a threshold value is exceeded, could enable an individually adapted response. For example, if temperature management is based on polystyrene boxes with additional cool or warm packs rather than automatic air conditioning, a QA system providing real-time alerts would be useful to enable a quick response to a critical deviation in temperature.

In contrast to temperature, the influencing effect of vibration emissions on spermatozoa is a relatively new topic. When using short-term extenders, vibrations are thought to cause a decrease in buffer capacity due to a loss of CO_2_ and thus an increase in pH, which in turn leads to a loss of sperm quality [26]. Further effects may be found at the molecular level. In industrial yogurt production, machine vibrations lead to undesirable texture changes due to the destruction of fine gel network strands [27] and the collision of protein particles [28]. These molecular interactions may also play a role in liquid preserved boar semen. Their role is currently being investigated by a number of research groups that are trying to determine the influence of vibration emissions depending on various factors [4,5]. Unfortunately, there is currently no consensus on the shaking frequencies with which the occurring vibrations should be simulated in the respective laboratories, making it difficult to set exact threshold values for harmful vibration emissions. For this purpose, this comprehensive analysis of occurring vibrations on German roads was carried out, using three-axis acceleration values. Unlike other studies that consider longitudinal, lateral, and vertical acceleration separately [6,15], the Displacement Index combines all three values to evaluate vibrations, since every movement, independent of its direction, can affect the liquid preserved boar semen. The general result of the standardized road trials is that both poorer road conditions and higher speeds lead to more vibrations. Surprisingly, the unpaved road showed lower vibrations than on cobblestone roads, which can be explained by the irregularity of individual impacts on dirt roads, whereas on cobblestones roads there are continuous strong vibrations. This could also be the reason why the highest measured $D_{\bar{x}}$-value on rough asphalt was measured at 30 km/h. Individual strong impacts are more prominent at lower speeds as they become blurred at higher speeds.

The vibration levels recorded during the 800 km German reference delivery are slightly higher compared to the data from the standard road trials on asphalted roads. This shows that the roads on this delivery are generally in good condition. However, factors of driving behavior such as acceleration, braking, or steering also influence occurring vibrations [29], and they could explain the slight increased displacement values. These factors were not considered during these standardized road trials, as this would have resulted in inconstant speed. In addition, it is plausible that the occurring vibrations are also influenced by the suspension of the vehicle used for delivery [30].

The data gathered during this study gave a good impression of the vibrations occurring during the transport of boar semen, and it allowed a more accurate simulation of laboratory tests to investigate the effect of vibrations on spermatozoa. In the future, when establishing threshold values for vibration emissions, the cumulative effect of intensity and duration of vibration exposure should be considered.

## 5. Conclusions

It is necessary to monitor parameters that may affect sperm quality during transport. Our main objective is to create a QA system consisting of a transport box with sensors for temperature, vibrations and light as well as a smartphone application. We suggest an Internet of Things (IoT) solution [17], using a smartphone application, which collects and processes measured data from one or more IoT-transport boxes and predicts the condition of the AI doses during transport in real-time. Optimally, the transport box will be air-conditioned as well as battery operated, in order to keep the temperature inside the box constant and the energy source independent of the vehicle. In case of an expected quality loss during transport due to exceeding predefined threshold values, the system should provide a real-time warning to the driver with recommended actions to handle the current situation. Using alternative routes would be one possibility to avoid high vibration emissions on spermatozoa, and the system could suggest appropriate detours to the driver. If there is no alternative approach to the customer, lowering the speed would most likely lead to decreasing vibration exposure. Even before the delivery vehicle is loaded, the system can support logistics by automatically creating delivery lists and optimizing routes in advance. As part of a QA system, the entire transport with all measured exposures is documented, especially time and temperature at the handover of the AI doses. If a problem occurs repeatedly on a particular section of the route, further targeted solutions could be found to lower harmful influences. Thus, the QA system could significantly contribute to a more stable quality in AI doses both in the short-term, through real-time alerts, as well as in the long-term, through optimized delivery routes.

## Figures and Tables

**Table 1:** Summary of data concerning boar semen shipping in Brazil, Germany, and USA.

Item	Characteristics
Semen packaging	tubes, blister
Logistics	employed drivers, subcontractors
Distances on average (range)	200 km (5–1500 km) (longer distances are covered by air freight)
Delivery duration on average (range)	4 h (0.25–12 h)
Customers per tour	1–30
Semen storage on transport	air-conditioned transportation car, air-conditioned box, polystyrene boxes with cool or warm packs
Monitored parameter	none, temperature, relative humidity
Handover to customer	semen storage unit on the farm, off farm drop location, semen depot

**Table 2:** Summary of vibration emissions during road trials (*n* = 120, 10 in each category) depending on road surface and speed. Several different routes were selected for measurements and each trial lasted *t* = 60 s. The speed kept constant with a cruise control.

Road Surface	Speed (km/h)	Mean $D_{\bar{x}}$	SD ($D_{\bar{x}}$)	CV ($D_{\bar{x}}$) (%)	Min (*D_i_*)	Max (*D_i_*)
(A) Roads with smooth asphalt	30	0.36	0.05	14.81	0.21	0.93
	60	0.46	0.02	5.08	0.22	1.27
	90	0.58	0.04	6.03	0.35	1.43
	120	0.74	0.05	7.02	0.43	1.82
	150	0.95	0.10	10.29	0.51	2.27
(B) Roads with rough asphalt	30	0.97	0.26	26.79	0.29	3.07
	60	0.80	0.07	8.41	0.30	3.36
	90	0.78	0.05	5.84	0.41	1.72
	120	0.86	0.06	7.03	0.55	1.93
(C) Cobblestone pavement	30	3.60	0.18	5.03	0.63	6.61
	60	4.44	0.09	2.06	1.74	7.76
(D) Dirt road	30	2.14	0.21	9.85	0.64	4.50

*D_i_*—Displacement Index in one second; $D_{\bar{x}}$—average Displacement Index (*D_i_*) over the entire trial (*t* = 60 s); Mean $D_{\bar{x}}$ summarizes 10 repetitions of test drives with standard deviation (SD) and coefficient of variation (CV); Min(*D_i_*), Max(*D_i_*)—minimum and maximum *D_i_* occurring in this category.

**Table 3:** Summarized results of two-way ANOVA, comparing mean Displacement Index $D_{\bar{x}}$ depending on speed and road surface.

Source	Degrees of Freedom	Sum Square	Mean Square	*F*-Value	*p*-Value
Speed	4	32.57	8.14	487.15	<0.001
Road surface	3	155.76	51.92	3106.68	<0.001
Speed:Road surface	4	3.26	0.81	48.76	<0.001
Residuals	108	0.02			

**Table 4:** Summarized results of the post hoc Tukey’s HSD test, comparing the mean Displacement Index $D_{\bar{x}}$ depending on road surface and speed (km/h).

Varying Factor	Group I	Group II	Mean Difference	*p*-Value	95% Family-Wise Confidence Levels	
					Lower Limit	Upper Limit
Speed	A-30	A-60	−0.10	0.877	−0.29	0.10
		A-90	−0.21	0.017 *	−0.41	−0.02
		A-120	−0.38	<0.001 ***	−0.57	−0.18
		A-150	−0.59	<0.001 ***	−0.78	−0.40
	A-60	A-90	−0.12	0.678	−0.31	0.08
		A-120	−0.28	<0.001 ***	−0.47	−0.09
		A-150	−0.50	<0.001 ***	−0.69	−0.30
	A-90	A-120	−0.16	0.201	−0.35	0.03
		A-150	−0.38	<0.001 ***	−0.57	−0.19
	A-120	A-150	−0.22	0.014 *	−0.41	−0.02
	B-30	B-60	0.17	0.141	−0.02	0.36
		B-90	0.19	0.065	−0.01	0.38
		B-120	0.11	0.769	−0.08	0.30
	B-60	B-90	0.02	> 0.999	−0.18	0.21
		B-120	−0.06	0.996	−0.26	0.13
	B-90	B-120	−0.08	0.967	−0.27	0.11
	C-30	C-60	−0.84	<0.001 ***	−1.04	−0.65
Road surface	A-30	B-30	−0.61	<0.001 ***	−0.42	−0.80
		C-30	−3.24	<0.001 ***	−3.05	−3.43
		D-30	−1.78	<0.001 ***	−1.59	−1.97
	B-30	C-30	−2.63	<0.001 ***	−2.44	−2.82
		D-30	−1.17	<0.001 ***	−0.98	−1.36
	C-30	D-30	1.46	<0.001 ***	1.65	1.27
	A-60	B-60	−0.34	<0.001 ***	−0.15	−0.54
		C-60	−3.99	<0.001 ***	−3.79	−4.18
	B-60	C-60	−3.64	<0.001 ***	−3.45	−3.84
	A-90	B-90	−0.21	0.024 *	−0.01	−0.40
	A-120	B-120	−0.13	0.579	0.07	−0.32

Road surface: A: smooth asphalt, B: rough asphalt, C: cobblestone, D: dirt road; * = *p* < 0.05, *** = *p* < 0.001.

**Table 5:** Summary of occurring Displacement Indices (*D_i_*) on a reference delivery from a German boar stud to several sow farms (distance = 800 km, *t* = 590 min).

Displacement Index (*D_i_*)	Frequency (%)	Absolute Time (Min)
≤1	32.9	194
>1–2	61.0	360
>2–3	4.6	27
>3–4	0.9	6
>4–5	0.4	2
> 5	0.1	1

## Data Availability

The data presented in this study are available on request from the corresponding author.
